# COVID-19 and Chaplains

**DOI:** 10.1177/1542305021994021

**Published:** 2021-04

**Authors:** Terry R. Bard

**Affiliations:** Editor in Chief, JPCP Inc

The 1918 H1N1 influenza A virus pandemic, the 1950’s Polio epidemic, and the Human Immunodeficiency Virus in the 1980s were all instructive about the likelihood that possible contagions would evolve. Steven Soderbergh’s 2011 film *Contagion* and Dustin T. Benson’s 2016 film *Pandemic* made the big screen but failed to penetrate the general human psyche. For many, the possibility of becoming infected with a lethal disease was simply theoretical yielding to denial. Few could imagine the virulence caused by a simple unseeable biological pathogen. After all, they could reason, instances of nasty viruses causing the so-called swine and bird flus were contained by scientific vigilance, and transmission was reasonably rapidly quelled.

Thus, when the SARS-CoV-2 (COVID-19) virus began to spread rapidly throughout the world in late 2019, most populations were unprepared for its lethal prevalence. What has followed in its wake has led to the death of millions, the challenges to scientists, economy and social devastation, and a reformulation of daily life. It is still raging even as a myriad of vaccines are becoming available. Beyond death and destruction, moral injury abounds and will continue even after this virus becomes tamed.

Without specific training for a pandemic, caregivers on the frontlines trying to help manage this disease and prevent deaths have been challenged in unanticipated ways, bringing many to the brink of their capacities. Chaplains and spiritual care providers have found themselves uniquely poised to provide support, guidance, and comfort to patients, families, and healthcare colleagues.

The *Journal of Pastoral Care & Counseling* has published several articles about chaplain experiences during this pandemic. Recently, in recognizing the global reach of its readership, the JPCP board of managers determined to make a formal effort to learn from chaplains and spiritual care providers world-wide. Angelika Zollfrank and Sophia Park began meeting with international colleagues to work on collaborative issues. The challenges of the current pandemic were quickly identified to be of concern for chaplains worldwide, and this group determined that a project to learn more about how chaplains were affected by the new and unprecedented demands.

This special issue inaugurates the 75th year of JPC&C continuous publication. It is edited by Anne Vandenhoeck and contains experiences, observations, and instruction from several international settings. We are pleased to offer it to our readership.

